# Temptation in the background: non-consummatory exposure to food temptation enhances self-regulation in boys but not in girls

**DOI:** 10.3389/fpsyg.2014.00788

**Published:** 2014-07-21

**Authors:** Aiste Grubliauskiene, Siegfried Dewitte

**Affiliations:** Faculty of Economics and Business, KU LeuvenLeuven, Belgium

**Keywords:** self-regulation, children, food, exposure to temptation

## Abstract

The abundance of calorie-dense low-nutrient food in everyday life raises the question as to how children deal with such opportunities. We investigate whether pre-exposure to the object of temptation in a situation that discourages consumption boosts children’s ability to resist similar temptation subsequently. We show that 7–12-year-old boys, but not girls, demonstrate increased resistance to a temptation after pre-exposure to a similar temptation. Future research might explore the role of exposure to temptation in girls.

## INTRODUCTION

Are sweets consumed in larger quantities if available at home? The intuitive answer is yes, and several studies confirm this intuition (Chandon and Wansink, 2002; Painter et al., 2002; Gorin et al., 2013). Contradicting this intuition, other research has shown that pre-exposure to food temptation can reduce subsequent consumption of a similar temptation, at least in adults, reflecting a mechanism of efficient self-regulation (Fishbach et al., 2003) that is based on change in preference (Geyskens et al., 2008). For children, one may argue that food availability may be more likely to induce subsequent consumption as they may lack efficient self-regulation strategies (Mischel and Underwood, 1974). Policy makers accordingly seem to assume that restricting access to unhealthy food is the best way to curb children’s overconsumption tendencies (Wardle, 1990).

However, prior findings suggest that the change in preference that is supposed to underlie the boosted subsequent resistance to temptation (Geyskens et al., 2008), one instantiation of self-regulation, may be more basic. Egan et al. (2007) demonstrated that children, similarly as adults, demonstrate cognitive dissonance by reducing the attractiveness of a previously rejected option (stickers). This suggests that children’s cognitive development may be advanced enough to also display the resistance to temptation after the pre-exposure to a similar temptation. We argue that exposure to temptation might boost rather than hurt subsequent resistance to temptation when the context of exposure discourages consumption. We consider a context of exposure to discourage consumption when a child autonomously assumes that consuming the tempting item is not desirable in that specific situation.

This paper addresses the question if exposing children to temptation in a context that discourages consumption enhances their subsequent resistance to a similar temptation, which has been documented in adults (Geyskens et al., 2008).

### EXPOSURE TO TEMPTATION AND RESISTANCE IN CHILDREN

A number of studies have demonstrated that food availability influences consumption (Hill and Peters, 1998). In the lab, food availability (Mischel and Ebbesen, 1970) reduced children’s willingness to wait for a larger food reward and promoted the choice of immediate smaller rewards. Field studies with 10–14-year-old children show that the availability of palatable foods at home is negatively associated with fruit and vegetable intake and positively associated with the consumption of soft drinks, sweets, and crisps (Vereecken et al., 2010).

Prior research on food exposure has conceptualized exposure to temptation without taking into account important boundary conditions. During pre-exposure to temptation in a situation in which free consumption is not appropriate (e.g., while waiting for other members of a party during a restaurant dinner) people seem to activate regulatory strategies to deal with the challenge. These strategies linger and when another similar tempting opportunity is subsequently presented, the successful strategies are more easily re-activated. This is theoretically consistent with prior research showing that exposure to food temptation without consuming it reduces adults’ desire and consumption in a subsequent tempting situation (Geyskens et al., 2008; Dewitte et al., 2009).

We argue that exposing children to temptation may boost rather than hurt subsequent resistance to temptation when the temptation is presented within a task context that discourages consumption (e.g., a word formation task with candy letters). In the face of temptation, this task goal corresponding to the word formation task would induce a goal conflict between the desire to consume the food and the situational inappropriateness of its consumption (e.g., a task cannot be accomplished if the candy is consumed). To solve this conflict, self-regulation – or conflict resolution – mechanisms will be activated. We argue that the activation of these conflict resolution mechanisms will facilitate subsequent resistance to temptation. We predict that children who are exposed to a temptation in a situation discouraging consumption will show increased resistance to a subsequent similar temptation, compared to children who are not pre-exposed to a temptation. At this point it is useful to note that, although superficially similar, our empirical focus differs from the delay-of-gratification paradigm in that these researchers were interested in resistance during the very exposure to temptation, whereas our interest is in measuring *subsequent* resistance to temptation after pre-exposure to temptation.

It is not clear whether primary school children would be able to solve a goal conflict between consumption and restriction in the face of a food temptation in a similar way as adults do. Some of the essential abilities for temptation control develop rather late. For instance, aiming to resist a temptation 7–11-year-old children have been shown to employ physical distraction strategies, such as covering their eyes, but they still do not spontaneously use cognitive distractions, such as changing the meaning of a temptation in their mind (Demetriou, 2000). Inhibition ability is still maturing at the age of 9 or even 12 (Williams et al., 1999; Leon-Carrion et al., 2004; Best et al., 2009; Best and Miller, 2010). Seven to eight-year-olds demonstrated a larger interference effect during a Stroop task than did adults, presumably because of their underdeveloped ability to actively inhibit distractors (Tipper et al., 1989). Interference suppression strategies in children might therefore not always be adequate. For example, they adopted a verbal strategy for a task (activate brain regions responsible for verbal processing) that was not inherently verbal (Bunge et al., 2002). The lack of self-regulation abilities in children might lead to succumbing to a temptation already during the first pre-exposure to temptation, in which resistance to temptation during the exposure is the essential feature.

In spite of these age-related procedural deficits in sophisticated self-regulation skills, we argue that pre-exposure to temptation in an involving context that effectively discourages consumption may help even children’s successful resistance to subsequent temptation. Recent research has shown that pairing positive pictures or palatable food with a no-go task lowered their evaluations (Veling et al., 2008, 2011a,b; Houben and Jansen, 2011). After making a choice, even 4-year-olds reliably decreased preferences for the rejected option (Egan et al., 2007), suggesting that the change in preference is less dependent on higher-level capacities, such as language, teaching, and socialization, than previously thought. In all these cases, learning that a stimulus was associated with inaction appeared to be easy, and it lingered to influence subsequent behavior. By associating tempting food (candy) with not responding (not eating, Van Osselaer, 2008) children might exercise momentary conflict resolution mechanisms which then linger and lead to resisting temptation in case another opportunity of consuming palatable food occurs. Thus a child is likely to benefit from pre-exposure to temptation if the situational cues induce a task goal that opposes the goal to consume the tempting item (e.g., completing a competitive task by using candies as objects). The focus of this research is to examine experimentally whether pre-exposure to food temptation is likely to enhance subsequent resistance to a similar temptation in children, based on our assumption that the conflict resolution mechanisms used during pre-exposure are still easily accessible.

## MATERIALS AND METHODS

### DESIGN AND PARTICIPANTS

This study utilized a unifactorial (pre-exposure to temptation: yes versus no) between-subjects design to test the effect of pre-exposure to temptation on children’s consumption behaviors.

Children aged between 7 and 12 years (*n* = 183), drawn from ten classes in two primary schools, were invited to participate in the study. Age was not significantly different between conditions. There were slightly more boys in the pre-exposure condition (54%) as compared to a control condition (49.2%). Participation was also contingent on children meeting the criteria of no history of behavioral or eating disorders. Informed consent from parents was acquired for 155 children (84%); as six children were absent at the time of the research, the final sample comprised 149 children. Two female experiment leaders conducted the experiment. Both experimenters collected data from both conditions.

### PROCEDURE

The study was completed individually and consisted of two phases: an exposure to temptation manipulation phase and a bogus taste test of a similar temptation.

Upon entering the school cafeteria children were greeted and asked for their name and age. Then, just before the exposure to temptation phase, their hunger state was measured on a visual three-point scale. This was achieved by presenting them with three cartoon drawings of children with their stomach showing different levels of hunger (Rolls et al., 2000). Then, the availability of the temptation was manipulated in the context of a 2-min word formation task. Each child was asked to use as many of 25 given letters as possible to form one or multiple words. During this task the experimenter was present at a distance in the same room. To induce a goal conflict, half of the children had to construct words from letter-shaped candy (pre-exposure condition). Children were not instructed not to eat candy. The experimenter observed unobtrusively whether the candies were eaten. None of children consumed the candy during this task. Another half of the children constructed words from cardboard letters without any candy present (no pre-exposure condition).

The second task involved a taste test, which was the same for all participants. Each participant was presented with two bowls, one with 100 g of regular *M&Ms*®; and another with 100 g of *Smarties*®;. Each child received a questionnaire about the candies, which included questions about several features of the candies (e.g., which is more delicious, *M&Ms* or *Smarties*?). Also included were questions about how much they liked *M&Ms* and *Smarties* on a three-point scale with three drawings of faces (frowning, neutral, and smiling). All children were allowed to taste as many of the *M&Ms* and *Smarties* as they desired. An experiment leader inconspicuously weighted the amount of candy left by each child.

Following standard practice, we considered the amount of candy consumed during the taste test as an indication of (a lack of) resistance to temptation. We expected that resistance to temptation in the pre-exposure to temptation condition would be better than resistance to temptation in a no pre-exposure to temptation condition. Additionally, we asked the teachers to provide the “good behavior” ratings, with higher numbers representing better behavior.

## RESULTS

Prior to analyses, 18 children were excluded. Four (5.9%) were lactose-intolerant, nine (13.4%) did not like the candies involved, and five (7.5%) did not properly perform the taste test as they ate none of the candy. The remaining sample consisted of 131 children (49% female). The consumption was log-transformed because its distribution was skewed. The effect of age was not significant, and is not discussed further. Since gender played a role, it was included in all analyses. The conditions differed neither in gender [*X*^2^(1) = 0.36, *p* = 0.55], nor in age [*F*(1,126) = 0.12, *p* = 0.73], nor in hunger level distribution [*F*(1,126) = 0.55, *p* = 0.46]. The “good behavior” ratings were higher in girls than in boys [*F*(1,126) = 10.73, *p* = 0.001], but these ratings neither influenced consumption (*r* = 0.02, *p* = 0.85), nor were they related to the consumption in each condition (pre-exposure condition: *r* = 0.14, *p* = 0.28; no pre-exposure condition: *r* = 0.18, *p* = 0.14).

**FIGURE 1:**
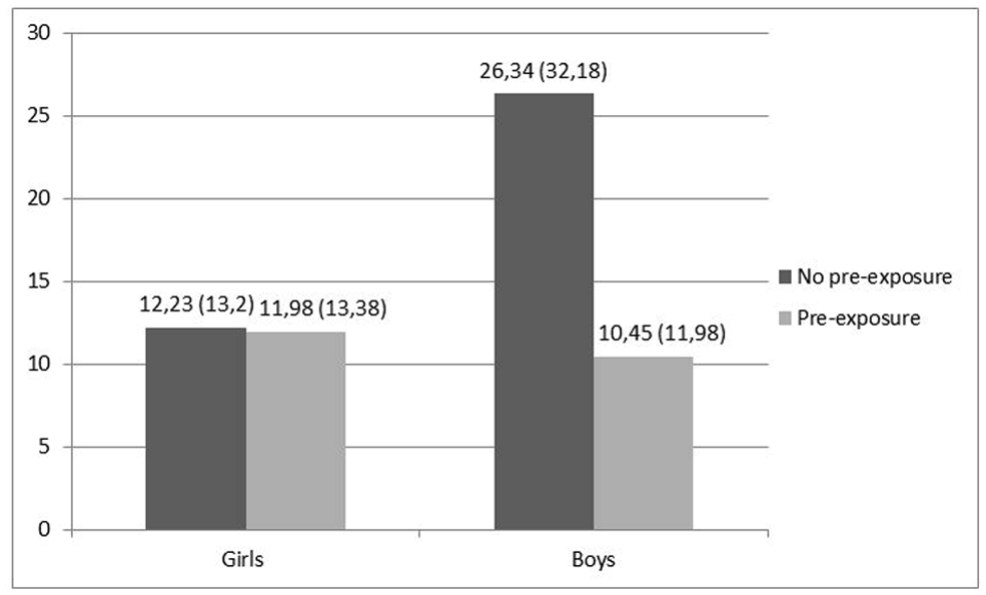
**The interaction of pre-exposure to temptation and gender on consumption (grams consumed)**.

To investigate resistance to temptation in children, a between-subject ANCOVA was used, with quantity of candy (*Smarties* and *M&Ms* combined, in grams) consumed during the taste test as the dependent variable, pre-exposure to temptation and gender as the independent variables, and hunger level as a covariate. Neither the main effect of gender [*F*(1,126) = 2.6, *p* = 0.11, $\eta_{p}^{2}$ = 0.02], nor that of pre-exposure on consumption [*F*(1,126) = 2.59, *p* = 0.11, $\eta_{p}^{2}$ = 0.02] was significant. There was a positive effect of hunger on consumption [*F*(1,126) = 6.38, *p* = 0.01, $\eta_{p}^{2}$ = 0.05]. The analysis revealed an interaction of pre-exposure to temptation and gender [*F*(1,126) = 7.02, *p* = 0.01, $\eta_{p}^{2}$ = 0.05; **Figure 1**]^1^. Simple effect analyses showed the expected effects of pre-exposure to temptation on consumption only for boys. For boys, the consumption after pre-exposure to temptation was lower than that in the no pre-exposure condition [*M* = 10.45 vs. 26.34; SD = 11.98 vs. 32.18; *F*(1,126) = 9.4, *p* < 0.003, *d* = -0.65]. For girls, the consumption after pre-exposure to temptation was not different from consumption in the no pre-exposure condition (*M* = 11.98 vs. 12.23; SD = 13.38 vs. 13.20; *F* = 0.52, *p* = 0.47, *d* = -0.02), being relatively low in both conditions. In pre-exposure condition, the consumption in boys was not different from the consumption in girls (*M* = 10.45 vs. *M* = 11.98, SD = 11.98 vs. 13.38, *F* = 0.55, *p* = 0.46, *d* = -0.12). In no pre-exposure condition, the consumption was higher for boys than for girls (*M* = 26.34 vs. 12.23, SD = 32.18 vs. 13.20, *F* = 8.77, *p* = 0.004, *d* = 0.57).

## DISCUSSION

The results offer experimental support for the hypothesis that exposure to calorie-dense, low-nutrient foods may facilitate resistance to temptation and hence ultimately have a beneficial effect on weight regulation in boys. The findings provide evidence that boys’ resistance to temptation increases after pre-exposure to temptation in situations where consumption is discouraged, thereby replicating findings in adults and suggesting that this type of self-regulation emerges early in life. The findings also question food policy assumptions (Wardle, 1990) that any exposure to tempting food cues always leads to overconsumption. Educators and parents could more effectively protect children against unhealthy food overconsumption by creating an environment that promotes the resistance to food by the children. For instance, boys might be offered to play a game where they can decide to save candy (which is present) in exchange for larger rewards later on (De Boer et al., 2014). However, the outcome should be interpreted cautiously because after exposure to temptation the resistance to temptation increased in boys, but not in girls.

Although the findings in boys are similar to the findings in adults in other studies, the conflict resolution mechanisms, leading to increased resistance, used by children might be different from the ones used by adults. For adults, a temptation often leads to the automatic activation of higher-priority goals, probably because of overlearning this association over the course of life (Fishbach et al., 2003, 2010; Papies et al., 2008). Children might not yet possess enough experience to have established a solid temptation – higher-priority goal association. Therefore for children solving a conflict might be more situational, present at the very concrete situation rather than relating to higher-priority goal. Future research could address the question on the nature of the conflict resolution mechanisms in children.

In this study, pre-exposure to temptation surprisingly did not influence resistance to temptation in girls. It is in contrast with other research which finds that girls typically react better to certain strategies by altering their eating-related behavior (Clark et al., 2007) as they are more than boys concerned about their weight status (Rolls et al., 1991). There are two possibilities why girls did not react to the pre-exposure manipulation. First, the interaction may reflect a real difference between genders because self-regulation strategies might have matured better in girls. This is consistent with the finding that girls’ self-regulation level without or with pre-exposure was similar to the boys’ self-regulation level after pre-exposure. In this sample, girls might already mastered their intake of tempting and unhealthy food without any external help and thus pre-exposure was not able to reduce their consumption which was low to begin with. However, it is not likely that girls of this age have already mastered food intake regulation to such an extent in the light of the finding that young adult females are still struggling with a response conflict regarding food and do react to pre-exposure to temptation (Geyskens et al., 2008).

Another possibility why girls did not alter their consumption may reside in the fact that the specific task distracted girls more than boys to such an extent that the goal conflict was reduced. During the task of pre-exposure to temptation, children were asked to form words from candy in the shape of letters, which requires attention and verbal skills. Being more skillful at focusing their attention to a specific target, more successfully ignoring tangential information (Pascualvaca et al., 1997), and being better in verbal tasks (Weiss et al., 2003), girls might have been more interested in the task and they might have completed that task more fluently than boys. Being more interested in the task, they might have not paid enough attention to the presented temptation (the candy letters) for the desire to consume them to occur. This could have prevented the conflict from appearing in the first place. Another possible explanation of difference in gender might be the level of compliance. As shown by a “good behavior” rating, girls generally tend to comply with the rules more than boys. Although these ratings did not influence the consumption during the taste test, this taste test could have been interpreted differently by girls and boys. However, the influence of the compliance is not straightforward. If girls had complied more during the taste test, they might have focused on the rule not to consume much (both in pre-exposure and no pre-exposure conditions), whereas boys, not caring much about the rules, might have consumed as much candy as they wanted, which was influenced by prior exposure to temptation. On the other hand, boys, not caring about the instructions, might have gorged with the candy in both conditions.

The study has a number of limitations. First, this is only one study demonstrating a better resistance to temptation after pre-exposure to a similar temptation in boys, but not in girls. Further studies with the same procedure and including gender as an independent variable should clarify whether the difference in gender that we find in this study is task specific, context specific (the experimenter leaders were both female), or more general. Further studies should look into whether studies with less gender-specific tasks and/or male experiment leaders result in the same outcome.

An interesting questions for future studies is which self-regulatory strategies are more active during pre-exposure – inhibiting the eating goal or activating the restriction goal. To solve a conflict during pre-exposure to temptation children might either concentrate more on completing the task or inhibit the urge to eat the presented candy. The answer to this question could provide valuable insights about children’s self-regulatory strategies in the presence of a temptation.

The interpretation of the findings that the self-regulatory strategies used during pre-exposure to temptation are still accessible during the second conflict with a similar temptation raises other questions for future research. One of them is whether these self-regulatory strategies would function in the same way if children were directly told not to consume the candy during the pre-exposure. If so, how strict should restrictions be? It could be that the direct request not to consume the candy will eliminate the need for self-regulatory strategies.

Another limitation of the present findings is that cognitive dissonance may be an alternative explanation of the effect. Per this account, less consumption by boys after not consuming the candy in the pre-exposure phase might be explained by their reduction of the preference of the candy upon their non-consumption. Not compatible with a cognitive dissonance explanation is the presence of a slightly different temptation in two tasks. Exactly the same items are usually used in a cognitive dissonance paradigm. However, we agree that we did not entirely rule out the cognitive dissonance account and it could be an interesting area of research for future studies. Based on the fact that self-regulation mechanisms are applied only when needed, the pre-exposure to temptation would reduce the preference for a temptation when there is an opportunity to consume in the second phase, but not – when there is no such opportunity. Based on cognitive dissonance account, the preference for a temptation should be reduced both when there is and there is no opportunity to consume that temptation in the second phase.

## Conflict of Interest Statement

The authors declare that the research was conducted in the absence of any commercial or financial relationships that could be construed as a potential conflict of interest.

## Footnotes

1 The results with the calories as dependent variable are similar, there is an interaction of pre-exposure to temptation and gender [*F*(1,126) = 7.38, *p* = 0.08, $\eta_{p}^{2}$ = 0.06].
